# Low-temperature redetermination of benzofurazan 1-oxide

**DOI:** 10.1107/S1600536809017036

**Published:** 2009-05-14

**Authors:** Seik Weng Ng

**Affiliations:** a Department of Chemistry, University of Malaya, 50603 Kuala Lumpur, Malaysia

## Abstract

In the six-membered ring of the low-temperature crystal structure of benzofurazan 1-oxide, C_6_H_4_N_2_O_2_, the two C atoms adjacent to the N atoms are linked by a delocalized aromatic bond [1.402 (2) Å]; each is connected to its neighbour by a longer, more localized, bond [1.420 (2), 1.430 (2) Å]. However, the next two bonds in the ring approximate double bonds [1.357 (2), 1.366 (2) Å]. As such, the six-membered ring is better described as a cyclo­hexa­diene system, in contrast to the description in the room-temperature structure reported by Britton & Olson (1979 ▶) [*Acta Cryst.* B**35**, 3076–3078].

## Related literature

For the room-temperature structure in the *P* 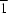 setting [6.772 (3), 7.515 (4), 7.759 (4) Å, 99.08 (3), 114.94 (3), 112.67 (3) °], see: Britton & Olson (1979 ▶). For the geometry-optimized structure, see: Friedrichsen, 1995 ▶; Ponder *et al.* (1994 ▶); Rauhut (1996 ▶). For details of the synthesis, see: Terrian *et al.* (1992 ▶); Wolthius (1979 ▶). For work mentioning the original structure, see: Ammon & Bhattacharjee (1982 ▶); Bird (1993 ▶); Cerecetto & González (2007 ▶); Ojala *et al.* (1999 ▶); Ramm *et al.* (1991 ▶).
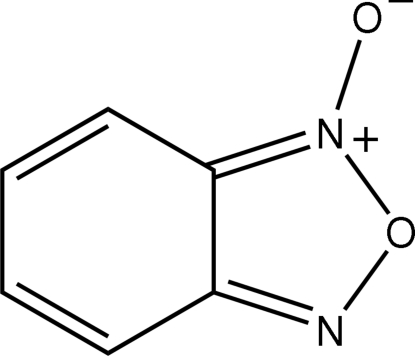


## Experimental

### 

#### Crystal data

C_6_H_4_N_2_O_2_
*M* *_r_* = 136.11
Triclinic, 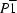

*a* = 6.6751 (2) Å
*b* = 7.3256 (2) Å
*c* = 7.6842 (2) Å
α = 100.710 (2)°
β = 114.265 (2)°
γ = 111.747 (2)°
*V* = 291.71 (1) Å^3^
*Z* = 2
Mo *K*α radiation
μ = 0.12 mm^−1^
*T* = 100 K
0.30 × 0.25 × 0.10 mm

#### Data collection

Bruker SMART APEX diffractometer
Absorption correction: none
1952 measured reflections
1276 independent reflections
1110 reflections with *I* > 2σ(*I*)
*R*_int_ = 0.012

#### Refinement

*R*[*F* ^2^ > 2σ(*F* ^2^)] = 0.035
*wR*(*F* ^2^) = 0.110
*S* = 1.03
1276 reflections
108 parameters
4 restraints
All H-atom parameters refined
Δρ_max_ = 0.33 e Å^−3^
Δρ_min_ = −0.21 e Å^−3^

### 

Data collection: *APEX2* (Bruker, 2008 ▶); cell refinement: *SAINT* (Bruker, 2008 ▶); data reduction: *SAINT*; program(s) used to solve structure: *SHELXS97* (Sheldrick, 2008 ▶); program(s) used to refine structure: *SHELXL97* (Sheldrick, 2008 ▶); molecular graphics: *X-SEED* (Barbour, 2001 ▶); software used to prepare material for publication: *publCIF* (Westrip, 2009 ▶).

## Supplementary Material

Crystal structure: contains datablocks I, New_Global_Publ_Block. DOI: 10.1107/S1600536809017036/tk2443sup1.cif

Structure factors: contains datablocks I. DOI: 10.1107/S1600536809017036/tk2443Isup2.hkl

Additional supplementary materials:  crystallographic information; 3D view; checkCIF report

## Figures and Tables

**Table 1:** Selected bond lengths (Å)

O1—N1	1.230 (1)
O2—N2	1.381 (1)
O2—N1	1.443 (2)
N1—C6	1.336 (2)
N2—C1	1.327 (2)
C1—C6	1.409 (2)
C1—C2	1.430 (2)
C2—C3	1.357 (2)
C3—C4	1.436 (2)
C4—C5	1.366 (2)
C5—C6	1.420 (2)

**Fig. 1.**
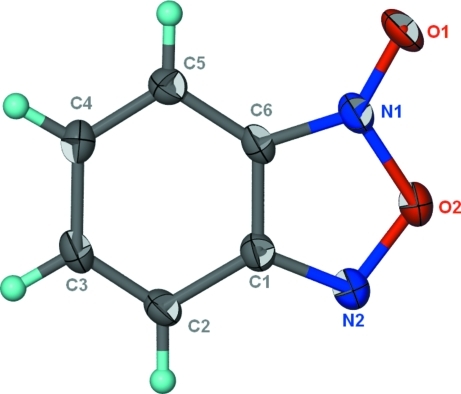
The molecular structure of the title compound. Displacement ellipsoids are drawn at the 70% probability level, and hydrogen atoms are drawn as spheres of arbitrary radius.

## supplementary crystallographic information

### Comment

Researchers have used the published structure of benzofurazan 1-oxide (Britton & Olson, 1979) in, for example, studies on packing (Ammon & Bhattacharjee, 1982; Ojala *et al.*, 1999; Ramm *et al.*, 1991), influence of *N*-oxide formation on heteroaromaticity (Bird, 1993), and reactivity and biology (Cerecetto & González, 2007). Bond dimensions from geometry-optimization calculations (Friedrichsen, 1995; Ponder *et al.*,1994; Rauhut, 1996) have also been compared with values taken from the solid-state structure.

The present low-temperature structure (Fig. 1 & Table 1) reveals features quite distinct from those disclosed in the original, room-temperature, analysis (Britton & Olson, 1979). In the six-membered ring, the two carbon atoms adjacent to the nitrogen atoms are linked by a delocalized aromatic bond [1.402 (2) Å]; each is connected to its neighbor by a longer, more localized, bond [1.420 (2), 1.430 (2) Å]. However, the next two bonds in the ring approximate double-bonds [1.357 (2), 1.366 (2) Å]. As such, the six-membered ring is better described as a cyclohexadiene system.

### Experimental

The compound was synthesized according to a reported procedure (Terrian *et al.*, 1992; Wolthius, 1979). Crystals were grown with THF as solvent.

### Refinement

The carbon-bound H-atoms were restrained to C—H 0.95±0.01 Å; their temperature factors were freely refined.

### Crystal data

**Table:**

C_6_H_4_N_2_O_2_	*Z* = 2
*M**_r_* = 136.11	*F*(000) = 140
Triclinic, *P*1	*D*_x_ = 1.550 Mg m^−^^3^
Hall symbol: -P 1	Mo *K*α radiation, λ = 0.71073 Å
*a* = 6.6751 (2) Å	Cell parameters from 1320 reflections
*b* = 7.3256 (2) Å	θ = 3.2–28.3°
*c* = 7.6842 (2) Å	µ = 0.12 mm^−^^1^
α = 100.710 (2)°	*T* = 100 K
β = 114.265 (2)°	Irregular block, yellow-orange
γ = 111.747 (2)°	0.30 × 0.25 × 0.10 mm
*V* = 291.71 (1) Å^3^	

### Data collection

**Table:**

Bruker SMART APEX diffractometer	1110 reflections with *I* > 2σ(*I*)
Radiation source: fine-focus sealed tube	*R*_int_ = 0.012
graphite	θ_max_ = 27.5°, θ_min_ = 3.2°
ω scans	*h* = −7→8
1952 measured reflections	*k* = −9→9
1276 independent reflections	*l* = −9→9

### Refinement

**Table:**

Refinement on *F*^2^	Secondary atom site location: difference Fourier map
Least-squares matrix: full	Hydrogen site location: inferred from neighbouring sites
*R*[*F*^2^ > 2σ(*F*^2^)] = 0.035	All H-atom parameters refined
*wR*(*F*^2^) = 0.110	*w* = 1/[σ^2^(*F*_o_^2^) + (0.0685*P*)^2^ + 0.0855*P*] where *P* = (*F*_o_^2^ + 2*F*_c_^2^)/3
*S* = 1.03	(Δ/σ)_max_ = 0.001
1276 reflections	Δρ_max_ = 0.33 e Å^−^^3^
108 parameters	Δρ_min_ = −0.21 e Å^−^^3^
4 restraints	Extinction correction: *SHELXL*, Fc^*^=kFc[1+0.001xFc^2^λ^3^/sin(2θ)]^-1/4^
Primary atom site location: structure-invariant direct methods	Extinction coefficient: 0.03 (1)

### Fractional atomic coordinates and isotropic or equivalent isotropic displacement parameters (Å^2^)

**Table:**

	*x*	*y*	*z*	*U*_iso_*/*U*_eq_	
O1	0.1183 (2)	0.2141 (2)	−0.0082 (1)	0.0274 (3)	
O2	0.5178 (2)	0.2611 (2)	0.1146 (1)	0.0271 (3)	
N1	0.3061 (2)	0.2329 (2)	0.1385 (2)	0.0211 (3)	
N2	0.7112 (2)	0.2839 (2)	0.2955 (2)	0.0257 (3)	
C1	0.6232 (2)	0.2701 (2)	0.4219 (2)	0.0188 (3)	
C2	0.7496 (3)	0.2863 (2)	0.6308 (2)	0.0202 (3)	
C3	0.6182 (3)	0.2669 (2)	0.7284 (2)	0.0207 (3)	
C4	0.3643 (3)	0.2321 (2)	0.6312 (2)	0.0209 (3)	
C5	0.2382 (3)	0.2174 (2)	0.4325 (2)	0.0199 (3)	
C6	0.3750 (2)	0.2377 (2)	0.3296 (2)	0.0181 (3)	
H2	0.919 (2)	0.310 (3)	0.693 (3)	0.030 (4)*	
H3	0.701 (3)	0.278 (3)	0.867 (2)	0.037 (5)*	
H4	0.281 (3)	0.221 (3)	0.706 (2)	0.027 (4)*	
H5	0.072 (2)	0.197 (3)	0.369 (2)	0.031 (4)*	

### Atomic displacement parameters (Å^2^)

**Table:**

	*U*^11^	*U*^22^	*U*^33^	*U*^12^	*U*^13^	*U*^23^
O1	0.0242 (5)	0.0333 (6)	0.0172 (5)	0.0139 (4)	0.0039 (4)	0.0130 (4)
O2	0.0270 (6)	0.0398 (6)	0.0190 (5)	0.0172 (5)	0.0137 (4)	0.0157 (4)
N1	0.0213 (6)	0.0237 (6)	0.0158 (5)	0.0105 (5)	0.0077 (5)	0.0098 (4)
N2	0.0237 (6)	0.0361 (7)	0.0202 (6)	0.0155 (5)	0.0120 (5)	0.0144 (5)
C1	0.0198 (6)	0.0199 (6)	0.0171 (6)	0.0096 (5)	0.0095 (5)	0.0090 (5)
C2	0.0187 (6)	0.0231 (6)	0.0175 (6)	0.0109 (5)	0.0072 (5)	0.0103 (5)
C3	0.0240 (7)	0.0215 (6)	0.0147 (6)	0.0109 (5)	0.0083 (5)	0.0093 (5)
C4	0.0246 (7)	0.0228 (6)	0.0192 (6)	0.0120 (5)	0.0137 (6)	0.0102 (5)
C5	0.0188 (6)	0.0213 (6)	0.0200 (6)	0.0104 (5)	0.0096 (5)	0.0096 (5)
C6	0.0200 (6)	0.0175 (6)	0.0136 (5)	0.0086 (5)	0.0066 (5)	0.0073 (4)

### Geometric parameters (Å, °)

**Table:**

O1—N1	1.230 (1)	C3—C4	1.436 (2)
O2—N2	1.381 (1)	C4—C5	1.366 (2)
O2—N1	1.443 (2)	C5—C6	1.420 (2)
N1—C6	1.336 (2)	C2—H2	0.956 (9)
N2—C1	1.327 (2)	C3—H3	0.948 (9)
C1—C6	1.409 (2)	C4—H4	0.946 (9)
C1—C2	1.430 (2)	C5—H5	0.947 (9)
C2—C3	1.357 (2)		
			
N2—O2—N1	109.4 (1)	N1—C6—C1	106.9 (1)
O1—N1—C6	136.0 (1)	N1—C6—C5	129.7 (1)
O1—N1—O2	117.7 (1)	C1—C6—C5	123.5 (1)
C6—N1—O2	106.3 (1)	C3—C2—H2	124 (1)
C1—N2—O2	105.0 (1)	C1—C2—H2	119 (1)
N2—C1—C6	112.5 (1)	C2—C3—H3	117 (1)
N2—C1—C2	128.0 (1)	C4—C3—H3	120 (1)
C6—C1—C2	119.5 (1)	C5—C4—H4	118 (1)
C3—C2—C1	116.8 (1)	C3—C4—H4	120 (1)
C2—C3—C4	122.9 (1)	C4—C5—H5	122 (1)
C5—C4—C3	121.9 (1)	C6—C5—H5	122 (1)
C4—C5—C6	115.4 (1)		
			
N2—O2—N1—O1	178.7 (1)	O1—N1—C6—C1	−178.3 (1)
N2—O2—N1—C6	−0.4 (1)	O2—N1—C6—C1	0.6 (1)
N1—O2—N2—C1	0.1 (1)	O1—N1—C6—C5	1.2 (2)
O2—N2—C1—C6	0.3 (2)	O2—N1—C6—C5	−179.9 (1)
O2—N2—C1—C2	−179.1 (1)	N2—C1—C6—N1	−0.5 (2)
N2—C1—C2—C3	179.9 (1)	C2—C1—C6—N1	178.9 (1)
C6—C1—C2—C3	0.6 (2)	N2—C1—C6—C5	179.9 (1)
C1—C2—C3—C4	−0.1 (2)	C2—C1—C6—C5	−0.7 (2)
C2—C3—C4—C5	−0.4 (2)	C4—C5—C6—N1	−179.2 (1)
C3—C4—C5—C6	0.3 (2)	C4—C5—C6—C1	0.2 (2)
